# Assessment of the implementation of SDG 4 goal by EU countries in the light of the 2030 Agenda using a hybrid approach in linear ordering

**DOI:** 10.1371/journal.pone.0333545

**Published:** 2026-06-22

**Authors:** Marek Walesiak, Grażyna Dehnel

**Affiliations:** 1 Department of Econometrics and Computer Science, Wroclaw University of Economics and Business, Wrocław, Poland; 2 Department of Statistics, Poznań University of Economics and Business, Poznań, Poland; Lucian Blaga University of Sibiu Faculty of Economics: Universitatea Lucian Blaga din Sibiu Facultatea de Stiinte Economice, ROMANIA

## Abstract

The goal of the following study was to rank EU countries on their progress in achieving SDG 4 “Ensure inclusive and equitable quality education and promote lifelong learning opportunities for all” in 2023 and to determine their distance from the target values set for 2030. Eurostat monitors and evaluates progress towards SDG 4 using six indicators. Based on these indicators, an aggregate measure was constructed according to the assumptions of a hybrid approach combining multidimensional scaling with linear ordering. The proposed method involves a non-compensatory approach and target-based data adjustment, which prevents high values of some indicators from offsetting shortfalls in others, thereby providing a more reliable assessment of progress towards policy targets. The results show that Nordic countries, along with the Netherlands and Estonia, rank highest, while Romania, Bulgaria, Cyprus and Greece remain the furthest from the 2030 targets. It is worth noting that no EU country has yet reached the aggregate SDG 4 target level, which highlights a substantial implementation gap at the EU level. The hybrid approach additionally makes it possible to visualise the multidimensional phenomenon in a two-dimensional space, enhancing interpretability of cross-country differences.

## 1. Introduction

The role of education in fostering economic growth and social development has been recognized since the origins of modern economic thought. Already in *The Wealth of Nations*, Adam Smith [1] emphasized that education enhances labour productivity and contributes to the wealth of nations by cultivating skills, discipline, and innovation capacity [2]. In the 20^th^ century, this idea evolved within the framework of human capital theory [3,4], which conceptualized education as an investment yielding both private and social returns. More recently, the empirical contributions of Abhijit Banerjee, Esther Duflo, and Michael Kremer have demonstrated that education not only drives economic efficiency but also reduces poverty and inequality by enabling more inclusive forms of growth [5,6].

In September 2015, 193 United Nations member states adopted the resolution “Transforming our world: the 2030 Agenda for Sustainable Development” containing 17 Sustainable Development Goals [7]. Within this context, Sustainable Development Goal 4 (SDG 4) “Ensure inclusive and equitable quality education and promote lifelong learning opportunities for all” is an important element of the 2030 Agenda for Sustainable Development. Given the significant differences between countries, including EU Member States, in education levels, quality, and participation in lifelong learning, systematic monitoring and review of SDG 4 implementation is required.

However, dominant approaches to monitoring SDG 4, typically based on composite indicators, face an important limitation. Most aggregation methods are compensatory, whereby high values of some indicators tend to offset the poor performance of others. In the context of SDG 4, this property may lead to misleading assessments, with some countries appearing to perform well overall despite failing to achieve key educational targets, which could lead to unfounded policy conclusions. At the same time, standard composite measures often are characterised by limited interpretability, as they offer rankings without clearly showing the distance from target values or the structure of multidimensional disparities.

The main research goal of the study was to rank the 27 EU countries according to their progress made in 2023 towards achieving SDG 4 goal “Ensure inclusive and equitable quality education and promote lifelong learning opportunities for all” and to determine their distance in relation to the target set for 2030.

To address the limitations of existing approaches, the study proposes a hybrid analytical approach combining multidimensional scaling (MDS) and linear ordering. This framework makes it possible not only to rank countries according to their overall progress toward SDG 4 but also to visualize their relative positions in a two-dimensional space, thereby enhancing the interpretability of multidimensional data. After selecting the optimal multidimensional scaling procedure presented in the methodological part the ranking of objects based on the optimal MDS procedure ensures the stability of the results. By incorporating target values and applying data adjustment, the method provides a more precise assessment of the distance to the target for each EU member state.

## 2. The characteristics of SDG 4 data

### 2.1. Objects, indicators, and target levels

The United Nations Goal 4 “Ensure inclusive and equitable quality education and promote lifelong learning opportunities for all” includes seven core targets, which are to be achieved by 2030 [8, pp. 5–6/24]. The global indicator framework contains 231 unique SDG indicators for 17 SDGs (thirteen indicators are used for two or three different targets). SDG 4 included in the UN publication contains 12 indicators [8]. Since 2017, Eurostat has published an “Annual EU SDG indicator review” (https://ec.europa.eu/eurostat/web/sdi/information-data), which contains an updated list of indicators for 17 SDGs (SDGs in the EU context). They are systematically monitored and assessed by Eurostat. Six indicators monitor progress on SDG 4. Four indicators from this list are partially consistent with the UN’s global indicator framework (see Table 1).

**Table 1:** Indicators and targets for SDG 4 for EU countries.

Eurostat code	Headline indicators	Code in the UN global list	Variable		Unit	EU 2030 target values	
			Type	Symbol			
Basic education							
04_40	Low achieving 15-year-olds in reading, mathematics or science	4.1.1 (s)	D	y1	%	15	(!)
04_31	Participation in early childhood education (the children between the age of three and the starting age of compulsory primary education)	4.2.2 (s)	S	y2	%	96	(!)
04_10	Early leavers from education and training (% of population aged 18–24)	4.1.2 (a)	D	y3	%	9	(!)
Tertiary education							
04_20	Tertiary educational attainment (% of population aged 25–34)	NA	S	y4	%	45	(!)
Adult learning							
04_60	Adult participation in learning in the past four weeks (% of population aged 25–64)	4.3.1 (s)	S	y5	%	26.22	(!!)
Digital skills							
04_70	Share of individuals having at least basic digital skills (% of individuals aged 16–74)	4.4.1 (s)	S	y6	%	80	(!!!)

(i) – identical, (s) – similar, (a) – alternative indicator.

S – stimulants (where higher values are more preferred), D – destimulants (where lower values are more preferred). Formal definitions can be found, e.g., in Hellwig [9, p. 48].

(!) – level set by the Resolution 2021/C 66/01 of the Council of the European Union [10].

(!!) – the level attained in 2023 by the top 10% of EU27 countries (a similar approach was proposed by OECD [11, p. 23] taking into account data for OECD countries).

(!!!) – Decision (EU) 2022/2481 of the European Parliament and of the Council [12].

Source: authors’ compilation based on data from Eurostat [13,14] (S1 File), Council of the EU [10] and the European Parliament and of the Council [12].

In this study, the analysis is intentionally based on the Eurostat SDG 4 indicator set, which ensures transparency, comparability, and policy relevance in the EU context. At the same time, it should be noted that these six indicators primarily capture quantitative and measurable aspects of education systems, such as participation, attainment, and skills, rather than the full conceptual scope of “quality education”. As a result, the study does not aim to provide a comprehensive assessment of educational quality, but rather to evaluate progress made by EU countries in relation to the Eurostat SDG 4 indicators and their associated 2030 target values. This distinction is important for the interpretation of results, as dimensions such as teaching quality, curriculum relevance, or socio-emotional competencies are not directly captured in the available data.

The following analysis is based on average data for the European Union and for 27 EU countries for the year 2023 (the Eurostat database: S1 File). The set of objects included 3 additional ones (S2 File): the target object with coordinates listed in Table 1 (EU 2030 target values), the pattern object (ideal; the upper pole) and the anti-pattern object (anti-ideal; the lower pole). Coordinates of the pattern object represent maximum values for stimulants and minimum values for destimulants (the most favourable values of preference variables), while the coordinates of the anti-pattern object represent minimum values for stimulants and maximum values for destimulants (the least favourable values).

Table 1 presents the indicators used in the analysis along with their target values. All statistical data come from the Eurostat database and are measured on a ratio scale. The selected variables include both stimulants (where higher values are preferred) and destimulants (where lower values are preferred), reflecting different dimensions of educational performance. It should be noted that the interpretation of some indicators requires caution. For example, indicators related to adult participation in learning and digital skills reflect not only educational outcomes but also broader structural characteristics, such as labour market organization, institutional settings, and digital infrastructure. Therefore, cross-country differences may partly reflect these contextual factors rather than education systems alone.

In the case of the variable “Low achieving 15-year-olds in reading, mathematics or science”, the relevant data come from 2022 and are derived from the PISA study (the Programme for International Student Assessment), which is a sample survey conducted every three years by the OECD. Owing to partial data unavailability, information about the variable “Participation in early childhood education” for Greece comes from 2019 and about the variable “Low achieving 15-year-olds in reading, mathematics or science” for Luxembourg – from 2018 (S3 File). These data constraints result from the periodicity and availability of international datasets and should be taken into account when interpreting the results.

Of the six variables shown in Table 1, the variable y3 (Early leavers from education and training) was achieved by as many as 16 countries in relation to the corresponding EU 2030 target of 9% in 2023. The EU target of 45% for the variable y4 (Tertiary educational attainment) was achieved by 13 countries, and the target of 96% for the variable y2 (Participation in early childhood education) was reached by 8 countries. Finland and the Netherlands achieved the target of 80% for the variable y6 (Share of individuals having at least basic digital skills), while Estonia and Ireland achieved the EU target of 15% for the variable y1 (Low achieving 15-year-olds in reading, mathematics or science). Denmark, the Netherlands, and Sweden achieved the target of 26.22% for the variable y5 (Adult participation in learning in the past four weeks).

Two countries managed to achieve four EU 2030 targets in 2023, namely Sweden (for the variables y2, y3, y4 and y5) and the Netherlands (for the variables y3, y4, y5 and y6), where as six countries achieved three targets: Belgium, France, Lithuania, Luxembourg, Poland (for the variables y2, y3 and y4) and Ireland (for the variables y1, y3 and y4). Bulgaria, Cyprus, Germany, Italy and Romania did not achieve any EU targets for 2030 in 2023.

### 2.2. The preparation of the dataset

Composite indicators (aggregate measures) play an important role in the analysis of socio-economic phenomena. The purpose of the following study is to rank EU countries in terms of their progress towards achieving SDG 4 in 2023 and determine their distance from the EU target values set for 2030. This assessment refers specifically to the Eurostat SDG 4 indicator set and should be interpreted as a measure of progress toward these operationalized targets, rather than a comprehensive evaluation of the overall quality of education systems. In order to achieve these objectives, the authors of the study applied an aggregate measure based on a hybrid approach involving multidimensional scaling combined with linear ordering. Ranking EU countries based on an aggregate measure requires the preparation of a dataset. We can distinguish in this regard three methodological approaches [15]:

1. Dataset does not contain the target object and data adjustment is not performed. The aggregate approach indicating the cumulative progress made by each EU country with respect to all indicators. Under this approach, it is not possible to determine the distance of each EU country from the EU 2030 target values. In the absence of data adjustment, the values of aggregate measures are distorted as a result of compensation. This approach was presented, for example, in the articles by Roszkowska and Wachowicz [16]; Szymańska and Zalewska [17].
2. Dataset contains the target object (with coordinates listed in Table 1) and data adjustment is not performed. The aggregate approach can be used to assess progress made by each EU country towards achieving SDG 4 and determine its distance from the 2030 target values. However, since data are not adjusted, values of aggregate measures are distorted as a result of compensation. This approach has been used, among others, by Miola and Schiltz [18]; OECD [11]; Lafortune et al. [19]; Roszkowska and Filipowicz-Chomko [20]; Tóthová and Heglasová [21].
3. The dataset contains the target object and data adjustment is performed. The aggregate approach can be used to assessing progress made by each EU country towards achieving SDG 4 and determining its distance from the 2030 target values. In addition in this methodological approach data adjustment is performed that eliminates the compensation effect by excluding higher values than the target values for stimulants and lower values than the target values for destimulants, which are replaced by EU 2030 target values (see step 4 in the procedure of the hybrid method in section 4.1). This approach was applied, for example, by Walesiak and Dehnel [15]; Becker et al. [22].

The main weakness of methods involving aggregate measures, as exemplified in the first and the second approach, is the fact they higher values offset the negative impact of lower values, which is known as the compensation effect. As a result, countries which have exceeded targets for some indicators but have failed to achieve those set for the majority of other indicators can be classified as countries that have made much progress towards SDG 4. To overcome the problems of the first and second approach, we applied an innovative third approach (S4 File), which accounts for the target values of the indicators and uses adjusted data (see section 4).

## 3. Literature review

This section of the study provides an overview of research regarding approaches to measuring progress towards SDG 4. The multidimensional nature of the SDGs – including SDG 4 (Quality Education) – requires the use of different methodological frameworks to adequately assess their implementation. The following review shows that some studies focus on primary data sources and rely mainly on qualitative methods, while others make use of secondary data and apply quantitative methods. At the same time, the literature indicates that the measurement of SDG 4 is not only a technical issue but also involves conceptual challenges related to how “quality”, “equity”, and “lifelong learning” are defined and operationalized in different socio-economic contexts.

The first group of studies includes Ferguson and Roofe [23] and Ferguson et al. [24], who analyse progress towards SDG 4 in high schools, identifying institutional challenges and opportunities for embedding sustainable development in curricula. They employ qualitative analysis, relying on stakeholder interviews and interpretative approaches. These studies stress the importance of subjective, context-rich assessments complementing indicator-based evaluations and argue that educational quality cannot be reduced to quantitative indicators alone, as it depends on institutional practices, teaching methods, and local contexts. Murdoch et al. [25] primarily relying on qualitative assessments, focus on the development and implementation of shared principles supporting the attainment of SDG 4 across European countries, emphasize governance mechanisms and coordination across education systems. Smith et al. [26] integrate both quantitative (logistic regression analysis) and qualitative (document analysis) methods to examine how countries prioritize SDG 4 indicators in the Voluntary National Reviews (VNRs). Their findings suggest that the selection and interpretation of indicators reflect national policy priorities, which introduces an additional layer of heterogeneity in SDG 4 monitoring.

A comprehensive assessment of progress towards achieving SDGs most often requires the use of secondary data sources, which offer a larger number of variables and enable cross-country comparisons. However, in the case of SDG 4, measurement involves specific challenges related to the interpretation of key dimensions. Indicators such as adult participation in learning or digital skills capture not only educational outcomes but also institutional arrangements of labour markets, welfare systems, and digital infrastructure. Similarly, measures of educational attainment or early school leaving may reflect both policy effectiveness and broader socio-economic inequalities. Consequently, the key research problem consists not only in selecting an appropriate method of multidimensional analysis, but also in ensuring that the aggregation procedure does not distort the meaning of these heterogeneous dimensions or lead to misleading policy conclusions. Table 2 provides an overview of studies based on secondary data related to SDG 4 or including SDG 4 as one of the analysed goals.

**Table 2:** Review of studies based on secondary data related to SDG 4 or including SDG 4 as one of the goals.

Study	Investigated SDGs (with SDG 4)	Applied method	Dataset characteristics	Data source
Studies measuring all or most SDGs including SDG 4				
ASVIS [27]	All SDGs	Composite indicators based on Adjusted Mazziotta-Pareto Index (AMPI)	28 EU countries, data from 2010 to at least 2015 (for SDG 4–2017), 88 indicators (6 indicators of SDG 4)	Eurostat
Miola and Schiltz [18]	All SDGs	Composite indicators based on: SDG Index, the OECD’s Distance measure, the Eurostat’s Progress measures	28 EU countries in 2018 and 145 indicators (6 indicators of SDG 4)	Eurostat, OECD, UN, WHO
OECD [11]	All SDGs	Composite indicators based on the OECD’s distance measure	36 OECD countries in 2018 and 132 indicators (9 indicators of SDG 4), time series data since 2005–2017 (76 indicators, 4 indicators of SDG 4)	UN, OECD
Hametner and Kostetckaia [28]	All SDGs	The compound average growth rate approach (CAGR) based on the Eurostat’s Progress measures and simple mean	28 EU countries and 99 indicators. Country data from the past 15 years for all indicators (with most recent data usually referring to 2017 or 2018). The number of indicators used in this study ranges from 5 indicators for SDG 17–11 indicators for SDG 3	Eurostat
Bie et al. [29]	All SDGs	Composite indicator based on the geometric average of the sustainability indexes (SI)	8 Arctic countries, 2000–2020 period, 69 indicators (5 indicators of SDG 4)	UN
Kuc-Czarnecka et al. [30]	16 SDGs (SDG 14 was excluded)	Composite indicator based on method of implementing tools derived from sensitivity analysis	27 EU countries in 2020, 75 indicators (5 indicators of SDG 4)	Eurostat
Qi et al. [31]	All SDGs	Composite Index Analysis Method based on Sachs et al. [32]	193 UN member States from 2010 to 2019, 67 indicators (2 indicators of SDG 4)	UN, the World Bank, FAO, WHO, UNICEF, OECD, and others
Blancas and Contreras [33]	All SDGs	Global sustainable development goals composite indicator (GSDGCI) that combines compensatory and non-compensatory aggregation rules	32 OECD countries, 95 indicators (4 indicators of SDG 4)	UN
Sachs et al. [34]	All SDGs	Composite indicator based on the SDG Index. The trend as the arithmetic average of all trend indicators	167 UN member States in 2019, 126 indicators with 102 global indicators and 24 indicators added for OECD countries (8 indicators of SDG 4). Trends 20152024 for 110 indicators	World Bank, OECD, WHO, FAO, ILO, UNICEF
Studies focused specifically on SDG 4				
Roszkowska and Filipowicz-Chomko [20, 35]	SDG 4	Composite indicator (classical and extended TOPSIS and Hellwig methods)	28 EU countries in 2015, 8 indicators	Eurostat, OECD
Szymańska and Zalewska [17]	SDG 4	Composite indicator (Hellwig method), cluster analysis, PCA	28 EU countries in 2018, 4 indicators	Eurostat
Saini et al. [36]	SDG 4	Exploratory Data Analysis (EDA)	India country dataset, 11 indicators. Yearly statistics for India from 1990 to 2020	the World Bank data repository, UNSTATS, UNESCO
Roszkowska and Wachowicz [16]	SDG 4	Composite indicator based on the entropy weight method and Hellwig method	27 EU countries in 2021 and 5 indicators	Eurostat

Source: own presentation.

A large group of studies measures all or most SDGs using composite indicators. ASVIS [27] developed composite indicators for all SDGs in the EU using the *Adjusted Mazziotta-Pareto Index (AMPI)*. 88 indicators were synthesized in this approach, including six related to education, to highlight disparities among 28 EU countries. The method enabled the use of multidimensional benchmarking but was limited by the availability and comparability of indicators across member states. Building on this foundation, Miola and Schiltz [18] expanded this perspective using a comparative framework combining the SDG Index, the OECD distance measure, and Eurostat progress metrics. The results of their analysis reveal a strong discrepancy between existing methods and suggest that a country’s relative position depends almost entirely on which method, and which indicators are selected. OECD [11] refined the *distance-to-target* approach for 36 OECD countries, incorporating nine education-related indicators. The study found that while access to education improved substantially, gaps in learning outcomes and lifelong learning opportunities persisted. The study included time-series data (2005–2017), enabling trend analysis that revealed slow but steady progress in educational equity and participation. Similarly, Hametner and Kostetckaia [28] employed the *compound annual growth rate (CAGR)* approach to assess the pace of SDG progress. Drawing on 15 years of data, their analysis showed substantial disparities in education performance among EU countries, pointing to differences in policy priorities and investment levels. Bie et al. [29] extended the analysis to Arctic countries, constructing a sustainability index based on the geometric mean of indicators. The inclusion of five SDG 4 indicators revealed that remote regions face unique challenges in ensuring education quality and access, often constrained by geography, demography, and digital infrastructure. Kuc-Czarnecka et al. [30] explored 16 SDGs (excluding SDG 14) using a *sensitivity analysis-based approach* across 27 EU countries. With five SDG 4 indicators, they examined relationships between education and other goals, identifying strong synergies with SDG 8 (decent work) and SDG 9 (innovation). Qi et al. [31] proposed a *Composite Index Analysis Method* based on Sachs et al. [32], assessing 193 UN member states across 67 indicators. Their methodological contribution lies in quantifying balance across indicators, providing an alternative lens to distance-from-target approaches by accounting for within-country disparities and uneven development patterns. With two education-related variables, this study highlighted inequalities in educational access and outcomes. Developed countries scored highly on access and completion rates, while developing nations faced persistent challenges in teacher training, resource allocation, and gender parity. Recent work by Blancas and Contreras [33] introduced the *Global Sustainable Development Goals Composite Indicator (GSDGCI)*, which combined compensatory and non-compensatory aggregation rules. By including four SDG 4 indicators, the authors demonstrated how education interacts with other social and economic dimensions, providing a more nuanced understanding of sustainability trade-offs. In parallel, Sachs et al. [34] in the *Sustainable Development Report 2025* presented global trends across 167 UN member states showing that despite progress in literacy and school enrollment, SDG 4 remains one of the slower-moving goals due to persistent inequities and pandemic-related disruptions. These studies emphasize that the methodological design of composite indicators—particularly the degree of compensation—has a direct impact on the interpretation of results.

Some studies focused entirely on SDG 4, employing specialized statistical and multi-criteria decision-making methods to explore educational performance and policy implications. Roszkowska and Filipowicz-Chomko [20, 35] developed composite indicators using the *TOPSIS* and *Hellwig* methods to measure educational sustainability in 28 EU countries. Using eight Eurostat and OECD indicators, they identified significant disparities between Northern and Western European countries, where education systems performed strongly, and Southern and Eastern Europe, where progress was slower. Their findings underscored the influence of the socio-economic context and policy frameworks on education outcomes. Szymańska and Zalewska [17] employed *Principal Component Analysis (PCA)* and *cluster analysis* based on four education indicators. Their results show that EU countries could be grouped into clusters according to their SDG 4 performance, closely mirroring broader socio-economic classifications. The study concluded that education outcomes are strongly correlated with indicators of social inclusion and innovation capacity. In a non-European context, Saini et al. [36] applied *Exploratory Data Analysis (EDA)* and a *genetic algorithm approach* to examine India’s progress on SDG 4 between 1990 and 2020. Analyzing 11 indicators, they identified patterns of association between gender parity, literacy rates, and higher education enrollment. Their findings indicate that digital expansion and gender equity significantly enhance education quality, demonstrating the potential of data-driven algorithms for national SDG monitoring. Roszkowska and Wachowicz [16] introduced methodological innovations by combining the *entropy weight* and *Hellwig methods* to evaluate education performance across 27 EU countries. Their analysis emphasized how normalization and weighting techniques can influence results, calling for greater transparency in index construction. This contribution is especially valuable for harmonizing methodologies across international SDG assessments.

The above review reveals that measuring progress towards SDG 4 involves both methodological and substantive challenges. On the one hand, composite indicators remain the dominant tool for aggregating multidimensional data. On the other hand, their compensatory nature may obscure structural weaknesses in specific dimensions of education, allowing high performance in one area to offset deficiencies in others. In the context of SDG 4, this issue is particularly important, as balanced progress across multiple dimensions—such as access, quality, and lifelong learning—is a core objective of the goal. As a result, compensatory aggregation may lead to misleading assessments and weaken the policy relevance of rankings.

Against this background, there is a need for methodological approaches that better reflect the target-based and multidimensional nature of SDG 4. The present study responds to this need by applying a hybrid approach combining multidimensional scaling with linear ordering and incorporating target-based data adjustment. This framework reduces the compensation effect and improves interpretability, thereby providing a more reliable basis for assessing progress towards SDG 4 in the European Union.

## 4. The research methodology

### 4.1. A hybrid approach to constructing an aggregate measure

A number of different approaches to constructing aggregate measures have been proposed in the literature depending on the degree of compensation [37, p. 3]. Studies on multi-criteria decision-making (see, e.g., [38–40]) uses of non-compensatory methods. An overview of compensatory and partially compensatory aggregate measures applied to different types of data can be found in [41,42].

**Fig 1:**
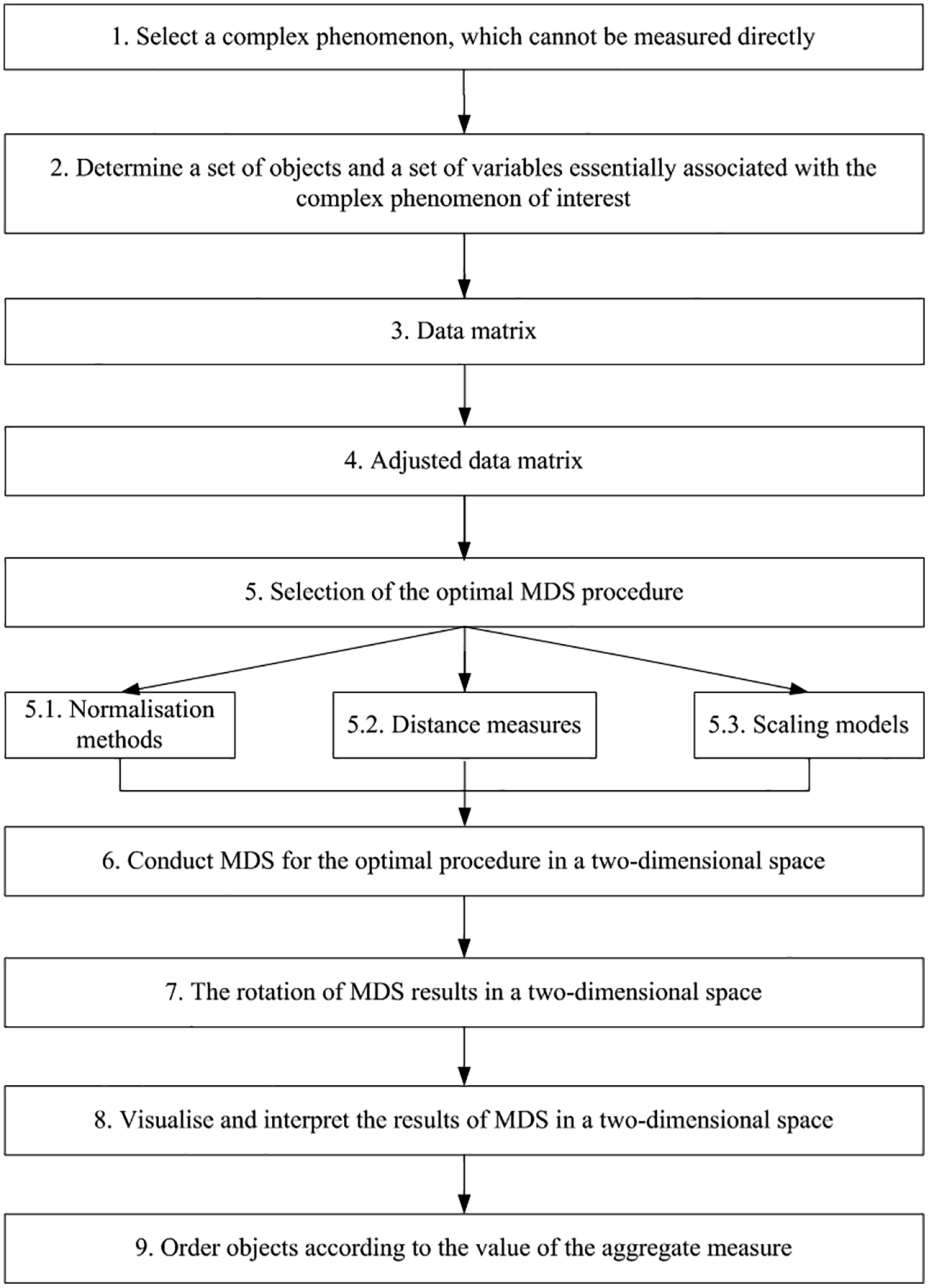
Steps in the procedure of the hybrid method. Source: own presentation.

The following study involves the hybrid method combining multidimensional scaling with linear ordering, which can be used to visualise results of linear ordering for metric data (see Walesiak, Dehnel and Dudek [43]). The procedure of the hybrid method consists of the following steps presented on Fig 1.

The first four steps of the hybrid method procedure include the preparation of the dataset. The hybrid method involves a combination of multidimensional scaling (steps 5–8) and linear ordering (step 9). After applying multidimensional scaling, objects of interest can be visualized in a two-dimensional space. Based on MDS results the objects are linearly ordered according to Hellwig’s [9] aggregate measure using the Euclidean distance from the pattern object.

The characteristics of the steps of the hybrid method in Fig 1 are shown in Table 3.

The method of constructing the aggregate measure by applying the hybrid approach makes it possible to:

- determine the distance of each EU country from the EU 2030 target;
- visualise results of linear ordering of objects in a two-dimensional space. This is not possible when other methods of linear ordering are used;
- enrich the visualisation by including isoquants of development (curves of equal development) and a development path (i.e., the shortest line connecting the development pattern and anti-pattern);
- use the functionality of the mdsOpt R package.

**Table 3:** Characteristics of the steps of the hybrid method.

Step	Characteristics / formulas	Source
1	Assessment of progress made by EU countries towards achieving SDG 4	NA
2	The study focused on 27 member states of the EU and the average object defined as the EU as a whole. The set of objects included 3 additional ones: the target object, the pattern object and the anti-pattern object. Progress towards SDG4 was assessed by taking into account 6 variables shown in Table 1.	NA
3	Observations for m=6 variables (from Table 1 describing SDG 4) for n+3=31 objects are arranged in the form of a data matrix: ${\left[y_{ij}\right]}_{(n+\text{3})\times m}=[\begin{array}{cccc} y_{\text{1}\text{1}} & y_{\text{1}\text{2}} & \cdots & y_{\text{1}m}\\ \vdots & \vdots & \cdots & \vdots \\ y_{n\text{1}} & y_{n\text{2}} & \cdots & y_{nm}\\ y_{T\text{1}} & y_{T\text{2}} & \cdots & y_{Tm}\\ y_{P\text{1}} & y_{P\text{2}} & \cdots & y_{Pm}\\ y_{AP\text{1}} & y_{AP\text{2}} & \cdots & y_{APm} \end{array}]$, where:i=1,…,n – object number (n=28: the EU and 27 EU countries), n+1=T – the target object, n+2=P – the pattern object, n+3=AP – the anti-pattern object, j=1,…,m – variable number.	NA
4	Observations for each variable are replaced by EU 2030 target values when the following conditions are met (for i=1,…,n): $x_{ij}=\left\{ \begin{array}{ccc} y_{Tj} & \text{for} & y_{ij}>y_{Tj}\\ y_{ij} & \text{for} & y_{ij}\leq y_{Tj} \end{array}\right.\;$, for stimulant $x_{ij}=\left\{ \begin{array}{ccc} y_{Tj} & \text{for} & y_{ij}<y_{Tj}\\ y_{ij} & \text{for} & y_{ij}\geq y_{Tj} \end{array}\right.\;$, for destimulant where:$y_{Tj}$ – EU 2030 target values for SDG 4 indicators, $x_{ij}$ – the *i*-th observation for the *j*-th variable.	Walesiak and Dehnel [15]
5	The optimal MDS procedure was selected by choosing from a combination of 9 normalizations methods, 4 distance measures and 4 MDS models (9×4×4=144 MDS procedures). Taking into account the two criteria (Kruskal’s Stress−1 measure of fit, the Hirschman-Herfindahl HHI index, calculated using Stress-per-point values) for all MDS procedures, for which ${Stress-\text{1}}_{p}\leq cs$ (cs – a midrange of Stress−1), we choose the one where $\underset{p}{\text{min}}\left\{{HHI}_{p}\right\}$ (p – MDS procedure number).	findOptimalSmacofSym function of mdsOpt R package (Walesiak and Dudek [44]), Borg et al. [45, p. 32], Herfindahl [46], Hirschman [47]
5.1	Normalize variable values (nine normalizations methods: n1, n3, n5, n5a, n8, n9, n9a, n11, n12a) and arrange them in the form of a normalized data matrix: $𝐙=[{z_{ij}]}_{(n+\text{3})xm}$ ($z_{ij}$ – normalized value of the *j*-th variable for the *i*-th object).	data.Normalization function from the clusterSim R package (Walesiak and Dudek [48])
5.2	Calculate the distance (distance measures for metric data: manhattan, Euclidean, squared Euclidean, GDM1), and arrange in the form of a distance matrix: $\delta ={\left[\delta_{ik}(𝐙)\right]}_{\left(n+\text{3})x(n+\text{3}\right)}$ (i,k=1,…,n,n+1,n+2,n+3).	See, e.g., Everitt et al. [49, pp. 49–50]; Jajuga et al. [50]
5.3	Four MDS models: ratio, interval, mspline model – polynomial function of the second and third degree.	the smacofSym function from the smacof R package (Mair et al. [51])
6	Conduct MDS for the optimal procedure ${f:\delta }_{ik}\left(𝐙\right)\to d_{ik}(𝐕)$ for all pairs (i,k), where *f* distance mapping from *m*-dimensional space $\delta_{ik}(𝐙)$ into corresponding distances $d_{ik}(𝐕)$ in a two-dimensional space to visualise relations between the analysed objects and to specify (interpret) the content of dimensions, which cannot be observed directly. They represent latent type of variables, which are used to explain similarities and differences between the analysed objects.	the smacofSym function from the smacof R package (Mair et al. [51]), Borg and Groenen [52, pp. 204–205])
7	Depending on the location of the pattern and anti-pattern object, the coordinate system needs to be rotated by an angle φ according to the formula: $[{{v^{\prime }}_{ij}]}_{(n+\text{3})x\text{2}}=[{v_{ij}]}_{(n+\text{3})x\text{2}}\times D$ ($[{{v^{\prime }}_{ij}]}_{(n+\text{3})x\text{2}}$ – a data matrix in a two-dimensional scaling space after rotating the coordinate system by an angle φ, $D=[\begin{array}{cc} os\varphi & -sin\varphi \\ sin\varphi & cos\varphi \end{array}]\;$– rotation matrix. The rotation does not change the arrangement of objects relative to one another but makes it possible to position the set axis connecting the pattern and anti-pattern along the identity line, which improves the visualization of results.	Bronsztejn et al. [53, p. 206]
8	This is done by joining two points representing the pattern and anti-pattern by a straight line to form what is known as the set axis in the diagram and drawing isoquants of development (curves of equal development) from the pattern. Objects located between the isoquants represent a similar level of development. The same level can be achieved by objects located at different points along the same isoquant of development (due to a different configuration of variable values).	NA
9	Order objects according to the value of the aggregate measure $d_{i}$ based on the Euclidean distance from the pattern object: $d_{i}=\text{1}-\frac{\sqrt{{\sum_{j=\text{1}}^{\text{2}}(v_{ij}-v_{+j})}^{\text{2}}}}{\sqrt{{\sum_{j=\text{1}}^{\text{2}}(v_{+j}-v_{-j})}^{\text{2}}}}$ ($v_{ij}$ – the *j*-th coordinate of the *i*-th object in the two-dimensional MDS, $v_{+j}(v_{-j})$ – the *j*-th coordinate of the pattern (anti-pattern) in the two-dimensional MDS. Values of the aggregate measure $d_{i}$ belong to the interval [0;1]. The higher the value of $d_{i}$, the higher the progress made by the analysed objects towards achieving SDG 4. The objects are arranged according to descending values of the aggregate measure $d_{i}$.	Hellwig [9]

Source: own presentation.

### 4.2. Selection of the optimal MDS procedure

The Stress measure (the goodness-of-fit statistic) is a summative index and does not inform how well a particular proximity value is represented in the given MDS space (Borg et al. [45], pp. 85–86). When evaluating MDS results we should take into account values of Stress-per-point (spp values) [45, p. 86; 54] and the Shepard diagram [55, 56]. When the distribution of errors associated with the location of individual objects in the scaling space (spp values) deviates considerably from the uniform distribution (e.g., the sum of errors for one or several objects exceeds 40%), the distribution of objects in a two-dimensional space is incorrect. In this case, the ranking of objects based on the results of multidimensional scaling in a two-dimensional space is distorted and does not reflect the actual situation (e.g., objects that should be located closer to the pattern object, are found closer to the anti-pattern object, while other objects may be located above the pattern object or below the anti-pattern object). Problems related to the use of multidimensional scaling in linear ordering are discussed in detail in the paper by Walesiak et al. [43]. The proposed solution in this case involves choosing the optimal multidimensional scaling procedure via application of the mdsOpt package, which offers two criteria: Kruskal’s Stress−1 (standardized residual sum of squares) measure of fit and the Hirschman-Herfindahl *HHI* index, calculated using Stress-per-point (spp) values. The problem of choosing the optimal scaling procedure (step 5 in Fig 1 and in Table 3) is presented in the paper Walesiak et al. [43].

**Fig 2:**
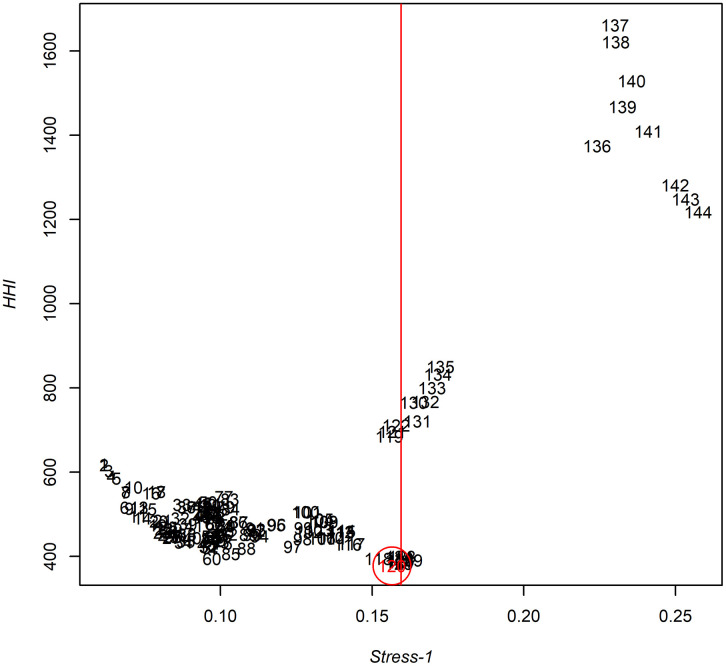
The values of *Stress*-1 fit measure and the *HHI* index for𝐩=144 MDS procedures (with the best solution denoted by the red circle). Source: The figure produced using the functionality of the package of R [57].

The optimal MDS procedure was selected by choosing from a combination of nine normalizations methods (implemented in the data.Normalization function from the clusterSim package: n1, n3, n5, n5a, n8, n9, n9a, n11, n12a), four distance measures (manhattan, Euclidean, squared Euclidean, GDM1) and four MDS models (ratio, interval, mspline model – polynomial function of the second and third degree). Fig 2 shows the relationship between Stress−1 and the HHI index, with the best solution denoted by the red circle (procedure 120), which satisfies the condition Stress−1≤a midrange of Stress−1 (0.1595435) and minimizes HHI. The Supporting Information section contains results for all MDS procedures. The ranking of objects based on the optimal MDS procedure provides the stability of the results.

Table 4 contains results for the best MDS procedure (120) after applying the hybrid method shown in Fig 1 (step 5).

**Fig 3:**
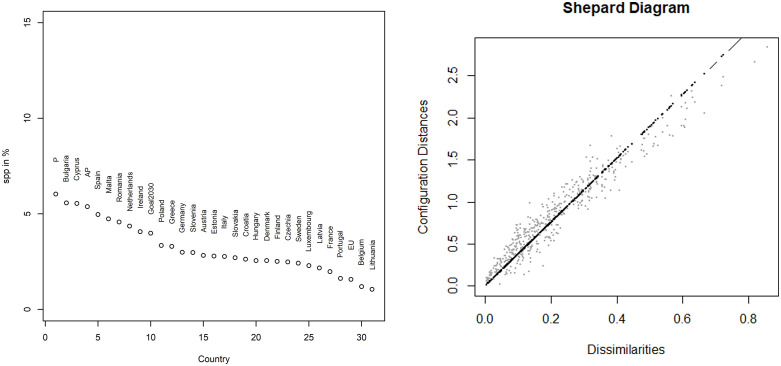
Stress plot and Shepard Diagram for MDS procedure 120. Source: own presentation using R [57].

Fig 3 show a Stress plot and a Shepard diagram for the optimal MDS procedure number 120. The distribution of errors (in terms of stress-per-point) does not deviate considerably from the uniform distribution (when the errors are uniformly distributed, the *HHI* index is equal to 322.58). The Shepard diagram offers a detailed insight into the goodness of fit between distances in the multidimensional space (Dissimilarities) and the scaling space (Configuration Distances). The diagram presents a general picture of dispersion around the function of regression, making it possible to detect outliers. In the optimal solution (see the Shepard diagram in Fig 3) there are no outliers.

**Table 4:** The results for the best MDS procedure (120).

Step in hybrid method (section 4)	Name	Best MDS procedure	STRESS-1	*HHI*
5.1	Normalization methods	n8	0.156543	377.35
5.2	Distance measures	GDM1		
5.3	Scaling models	ratio		

n8 – $z_{ij}=\frac{x_{ij}}{\underset{i}{max}\{x_{ij}\}}$;

GDM1 – $\delta_{ik}=\frac{\text{1}}{\text{2}}-\frac{\sum_{j=\text{1}}^{m}\left(z_{ij}-z_{kj}\right)\left(z_{kj}-z_{ij}\right)+\sum_{j=\text{1}}^{m}\sum_{\begin{array}{c} =\text{1}\\ l\neq i,k \end{array}}^{n}\left(z_{ij}-z_{lj}\right)\left(z_{kj}-z_{lj}\right)}{\text{2}{\left[\sum_{j=\text{1}}^{m}\sum_{l=\text{1}}^{n}{\left(z_{ij}-z_{lj}\right)}^{\text{2}}\cdot \sum_{j=\text{1}}^{m}\sum_{l=\text{1}}^{n}{\left(z_{kj}-z_{lj}\right)}^{\text{2}}\right]}^{\text{0},\text{5}}}$;

i,k,l=1,…,n *–* object number, j=1,…,m *–* variable number; *m* – number of variables, $x_{ij}\left(x_{kj},x_{lj}\right)$ – *i*-th (*k*-th, *l*-th) observation for the *j*-th variable; $z_{ij}\left(z_{kj},z_{lj}\right)$ – normalised value of *j*-th variable for the *i*-th (*k*-th, *l*-th) object.

Source: own presentation using the mdsOpt package of R [57].

## 5. Results in relation to EU-level targets for 2030

**Fig 4:**
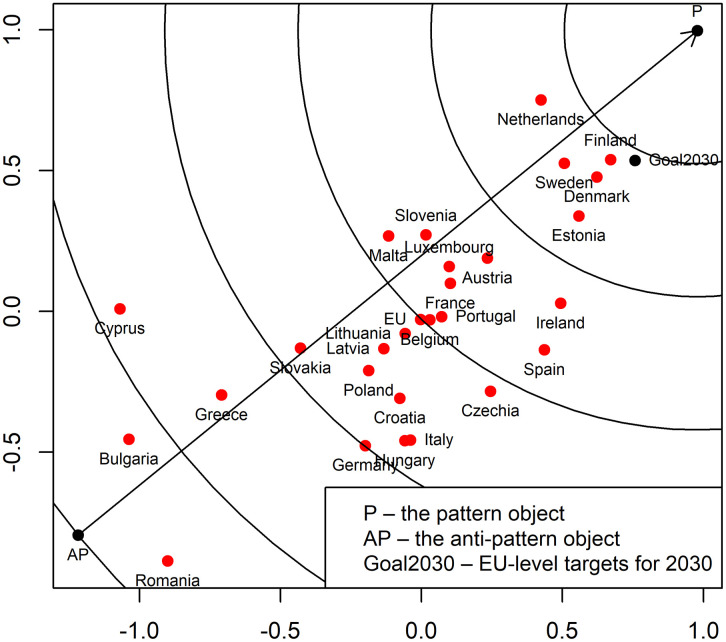
Visualisation of multidimensional scaling results in a two-dimensional space of progress made by EU countries in 2023 towards SDG 4. Source: author’s compilation using R [57].

Fig 4 shows the results of multidimensional scaling of 31 objects – 27 EU countries, the EU, the 2030 EU target, the pattern object (P) and the anti-pattern object (AP) – representing progress made by EU countries towards achieving SDG 4 in the light of the 2030 Agenda. The use of multidimensional scaling in the hybrid approach allowed for the visualisation in a two-dimensional space the degree to which 27 EU countries managed to achieve the EU 2030 targets for SDG 4 taking into account 6 indicators listed in Table 1. The pattern (P) and the anti-pattern object (AP) were connected with a straight line to form what is known as the set axis. Six isoquants of development divide the set axis into equal parts. EU countries located between each pair of isoquants represent a similar level of progress towards SDG 4. EU countries located at different points on the same isoquant of development have the same level of progress, which results from different combinations of indicator values. The further away from the pattern object of development a given isoquant band is, the less progress EU country located in that band made towards achieving SDG 4.

Taking into account the configuration of objects in a two-dimensional (Fig 4), values of the aggregate measure $d_{i}$ were calculated according to the aggregate measure $d_{i}$. Results of linear ordering of 27 EU countries in terms of their progress in 2023 towards achieving SDG 4 ($d_{i\text{2}\text{0}\text{2}\text{3}}$) and the distance of each country from the EU 2030 target ($\Delta_{i}=d_{i\text{2}\text{0}\text{2}\text{3}}-d_{i\text{2}\text{0}\text{3}\text{0}}$) are shown in Table 5. The higher the value of $d_{i}$, the more progress a given country made in 2023 towards achieving SDG 4. The higher the value of $\Delta_{i}$, the closer a given country is towards achieving SDG 4 in 2030.

The validity of the third approach was confirmed by the application of the procedure described in section 4 for unadjusted data (the second approach). The resulting values of the aggregate measure $d_{i\text{2}\text{0}\text{2}\text{3}}$ incorrectly suggest that Sweden, the Netherlands exceeded the 2030 target for SDG 4, while, in fact, neither Sweden nor the Netherlands achieved all 2030 targets for 2030 (see the last paragraph in section 2.1). Sweden did not achieve the targets for “Low achieving 15-year-olds in reading, mathematics or science” and “Share of individuals having at least basic digital skills”, while the Netherlands did not achieve the targets for “Low achieving 15-year-olds in reading, mathematics or science” and “Participation in early childhood education”.

**Table 5:** Results of linear ordering of EU countries in terms of progress they made in 2023 towards achieving SDG 4.

Symbol	Country	$d_{i2023}$	Rank	$\Delta_{i}$	Class	Level
FI	Finland	0.8053	1	−0.0143	1	Very high
NL	Netherlands	0.7861	2	−0.0335		
DK	Denmark	0.7777	3	−0.0419		
SE	Sweden	0.7648	4	−0.0549		
EE	Estonia	0.7246	5	−0.0950		
IE	Ireland	0.6181	6	−0.2015	2	High
AT	Austria	0.6124	7	−0.2072		
SI	Slovenia	0.5750	8	−0.2446	3	Middle
LU	Luxembourg	0.5713	9	−0.2483		
FR	France	0.5575	10	−0.2621		
ES	Spain	0.5567	11	−0.2629		
MT	Malta	0.5359	12	−0.2837		
PT	Portugal	0.5196	13	−0.3000		
BE	Belgium	0.5066	14	−0.3130		
EU	European Union	0.4991	15	−0.3206		
CZ	Czechia	0.4793	16	−0.3403		
LT	Lithuania	0.4730	17	−0.3466		
LV	Latvia	0.4407	18	−0.3789		
PL	Poland	0.4079	19	−0.4117		
HR	Croatia	0.4079	20	−0.4117		
IT	Italy	0.3741	21	−0.4455	4	Weak
HU	Hungary	0.3692	22	−0.4504		
SK	Slovakia	0.3638	23	−0.4559		
DE	Germany	0.3346	24	−0.4851		
EL	Greece	0.2497	25	−0.5699	5	Very weak
CY	Cyprus	0.1978	26	−0.6218		
BG	Bulgaria	0.1235	27	−0.6961		
RO	Romania	0.0614	28	−0.7582		

$\Delta_{i}$ – country’s distance from the EU 2030 target; $\Delta_{i}=d_{i\text{2}\text{0}\text{2}\text{3}}-d_{i\text{2}\text{0}\text{3}\text{0}}=d_{i\text{2}\text{0}\text{2}\text{3}}-\text{0}.\text{8}\text{1}\text{9}\text{6}$; $mean\left(d_{i\text{2}\text{0}\text{2}\text{3}}\right)=\text{0}.\text{4}\text{8}\text{9}\text{1}$; $sd\left(d_{i\text{2}\text{0}\text{2}\text{3}}\right)=\text{0}.\text{1}\text{8}\text{9}\text{3}$; $mean\left(\Delta_{i}\right)=-\text{0}.\text{3}\text{3}\text{0}\text{6}$; $sd\left(\Delta_{i}\right)=\text{0}.\text{1}\text{8}\text{9}\text{3}$

Source: Calculated using the R [57].

The typological classes in Table 5 (last column) were defined in accordance with the mean (mean) and standard deviation (sd) approach (see Table 6). Identical results regarding the typological classes are obtained by substituting values of $d_{i\text{2}\text{0}\text{2}\text{3}}$, $mean\left(d_{i\text{2}\text{0}\text{2}\text{3}}\right)$ and $sd\left(d_{i\text{2}\text{0}\text{2}\text{3}}\right)$ with values of $\Delta_{i}$, $mean\left(\Delta_{i}\right)$ oraz $sd\left(\Delta_{i}\right)$, respectively.

**Table 6:** The typological classes according to the level of progress towards SDG 4.

Class	Criterion	Level
1	$d_{i\text{2}\text{0}\text{2}\text{3}}>mean\left(d_{i\text{2}\text{0}\text{2}\text{3}}\right)+sd\left(d_{i\text{2}\text{0}\text{2}\text{3}}\right)$	very high
2	$mean\left(d_{i\text{2}\text{0}\text{2}\text{3}}\right)+\text{0}.\text{5}\cdot sd\left(d_{i\text{2}\text{0}\text{2}\text{3}}\right)<d_{i\text{2}\text{0}\text{2}\text{3}}\leq mean\left(d_{i\text{2}\text{0}\text{2}\text{3}}\right)+sd\left(d_{i\text{2}\text{0}\text{2}\text{3}}\right)$	high
3	$mean\left(d_{i\text{2}\text{0}\text{2}\text{3}}\right)-\text{0}.\text{5}\cdot sd\left(d_{i\text{2}\text{0}\text{2}\text{3}}\right)<d_{i\text{2}\text{0}\text{2}\text{3}}\leq mean\left(d_{i\text{2}\text{0}\text{2}\text{3}}\right)+\text{0}.\text{5}\cdot sd\left(d_{i\text{2}\text{0}\text{2}\text{3}}\right)$	middle
4	$mean\left(d_{i\text{2}\text{0}\text{2}\text{3}}\right)-sd\left(d_{i\text{2}\text{0}\text{2}\text{3}}\right)<d_{i\text{2}\text{0}\text{2}\text{3}}\leq mean\left(d_{i\text{2}\text{0}\text{2}\text{3}}\right)-\text{0}.\text{5}\cdot sd\left(d_{i\text{2}\text{0}\text{2}\text{3}}\right)$	weak
5	$d_{i\text{2}\text{0}\text{2}\text{3}}\leq mean\left(d_{i\text{2}\text{0}\text{2}\text{3}}\right)-sd\left(d_{i\text{2}\text{0}\text{2}\text{3}}\right)$	very weak

Source: own presentation.

It is worth noting that in 2023 as many as 13 countries achieved a similar level of progress, represented by the average level of the aggregate measure, ranging from $d_{i\text{2}\text{0}\text{2}\text{3}}=\text{0}.\text{4}\text{0}\text{7}\text{9}$ (Croatia) to $d_{i\text{2}\text{0}\text{2}\text{3}}=\text{0}.\text{5}\text{7}\text{5}\text{0}$ (Slovenia). This class includes the average EU object.

**Fig 5:**
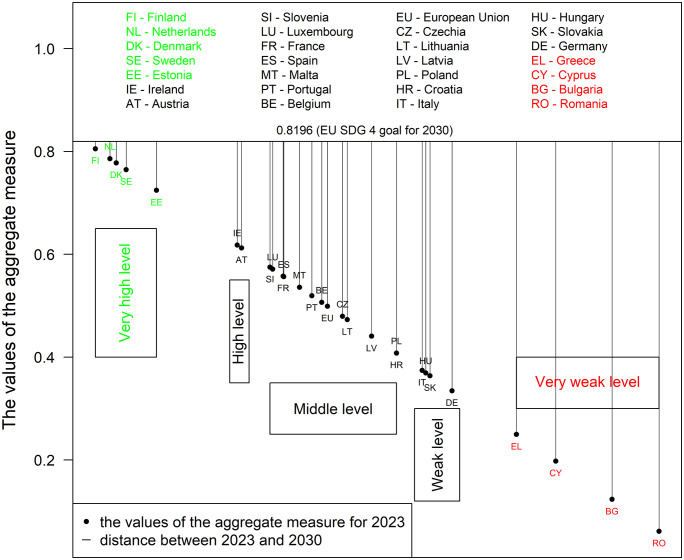
A graphical representation of distances separating EU countries from the EU target for 2030. Source: chart created using R [57].

The distance of particular countries from the EU 2030 target ($\Delta_{i}=d_{i\text{2}\text{0}\text{2}\text{3}}-d_{i\text{2}\text{0}\text{3}\text{0}}$) is visualised in Fig 5. The horizontal line represents the EU-level SDG 4 target value of the aggregate measure $d_{i\text{2}\text{0}\text{3}\text{0}}=\text{0}.\text{8}\text{1}\text{9}\text{6}$.

None of the EU countries achieved this target value although Finland came the closest. The top five countries included the Scandinavian countries, the Netherlands and Estonia (in Fig 5 marked in green). The distance of the European Union as a whole in 2023 in relation to the 2030 target is Δ=0.3206. Countries that came close to the EU average include Belgium, Portugal, Malta, Czechia and Lithuania. Fourteen EU countries were closer to the target in 2023 than the EU average (see Fig 5). Romania, Bulgaria, Cyprus and Greece were the furthest away from the 2030 target (in Fig 5 marked in red).

To complement the ranking-based interpretation, selected country profiles are presented to illustrate how similar aggregate results can result from different indicator configurations:

- High performers (Finland, Netherlands): Finland, ranked first, achieved the target value for only one indicator (Digital skills). Nonetheless, the country did not fully meet all the other targets of SDG 4, which explains why it still remains below the EU 2030 benchmark. Its profile reflects a balanced and consistently strong performance across different dimensions, in contrast to that of the Netherlands, which ranked second. While it achieved target values for four indicators, including tertiary education and early school leaving, it is still far from the EU targets with regard to one indicator (Low achieving 15-year-olds).
- Middle performers (Poland, Croatia): Poland achieved selected targets (particularly for early childhood education, early school leaving, and higher education). However, it performed significantly worse in terms of participation in adult education and digital skills. Croatia only met the target for one indicator (early school leaving), but is significantly closer to meeting the target for digital skills indicator than Poland.
- Low performers the bottom three countries (Romania, Bulgaria, Cyprus) in the ranking have not achieved any of the EU 2030 targets in 2023. Their poor position reflects persistently low values across a number of indicators, most notably low achieving 15-year-olds and digital skills indicators.

These examples confirm that countries located on the same or adjacent isoquants may reach similar aggregate scores through different combinations of indicator values. Therefore, the results should be interpreted not only in terms of ranking positions but also in terms of underlying indicator structures, which are essential for identifying policy priorities.

## 6. Analysis of the sensitivity and robustness of the hybrid method

The sensitivity and robustness of the hybrid method was assessed by conducting an additional analysis, in which one or two of the target variables were excluded. The results of linear ordering based on the full and reduced set of variables were assessed using Spearman’s rank correlation coefficient are presented in Tables 7 and 8.

**Table 7:** The similarity of rankings in terms of values of Spearman’s rank correlation coefficient (sensitivity analysis with the exclusion of one variable).

Ranking based on all variables	Rankings with the exclusion of one variable					
	y1	y2	y3	y4	y5	y6
y1-y6	0.9704	0.9978	0.9863	0.9847	0.8922	0.9655

Source: calculated using the R [57].

**Table 8:** The similarity of rankings in terms of values of Spearman’s rank correlation coefficient (sensitivity analysis with the exclusion of two variables).

Rankings with the exclusion of two variables	Ranking based on all variables y1-y6
y1, y2	0.9644
y1, y3	0.9458
y1, y4	0.9584
y1, y5	0.8276
y1, y6	0.9332
y2, y3	0.9869
y2, y4	0.9836
y2, y5	0.8752
y2, y6	0.9693
y3, y4	0.9704
y3, y5	0.8451
y3, y6	0.9660
y4, y5	0.8281
y4, y6	0.9584
y5, y6	0.6278

Source: calculated using the R [57].

The results in Table 7 indicate that the application of the hybrid approach is not greatly affected by the exclusion of one variable (values of Spearman’s ρ ranging from 0.8922 to 0.9978). Similarly, the hybrid approach is not much affected the exclusion of two variables, as indicated by the values of Spearman’s ρ ranging from 0.8276 to 0.9869 (Table 8), with the exception of variables y5 and y6, where ρ is considerably lower (0.6268).

The hybrid approach is not seriously affected by missing data in y1 for Luxembourg and in y2 for Greece, as mentioned in subsection 2.1. Even when both variables are excluded from the analysis, ρ is equal to 0.9644 (Table 8).

A sensitivity analysis across normalization choices, distance metrics, and MDS procedures (Supporting information – S1 Table) is a complex problem, which requires a broader discussion and presentation. It turns out that MDS procedures with higher values of the *HHI* index do not always lead to a considerable change in the ranking of objects. The results may be distorted by the concentration or dispersion of the values of the aggregate measure used to conduct linear ordering. Section 3 of article [43] reports examples of significant ranking changes for MDS procedures with high values of the *HHI* index. In the hybrid approach linear ordering is preceded by multidimensional scaling in a two-dimensional space. This step causes the loss of information resulting from the shift of the original configuration of objects in a multidimensional space (defined in article by six variables) to a two-dimensional space. Consequently, in the case of these procedures, MDS results in a two-dimensional space must be analysed in terms of the distribution of errors associated with the configuration of particular objects. In the ideal case, the distribution of errors should be uniform (each object should be arranged in a two-dimensional space with the same error). The more the errors of MDS results deviate from the uniform distribution, the worse the results of linear ordering are. This analysis can be facilitated by box plots.

To illustrate the complexity of the problem, the authors analysed ten selected from S1 Table procedures: two procedures of with the best values of the *HHI* index (no. 120 and 123), and two with the worst values (no. 138 and 137), two with the best values of Stress (no. 2 and 1), two (no. 30 and 99) with the highest and lowest value of Spearman’s ρ with the optimal MDS procedure, and two intermediate ones (no. 100 and 59). Table 9 shows values of Spearman’s ρ between rankings obtained with procedure 120 and the above selected procedures.

**Table 9:** The similarity of rankings in terms of values of Spearman’s rank correlation coefficient.

Ranking based on procedure	Rankings based on procedures								
	123	30	99	100	59	2	1	138	137
120	0.9887	0.9980	0.9315	0.9762	0.9827	0.9827	0.9827	0.9770	0.9766

Source: calculated using the R [57].

Values of Spearman’s ρ between the ranking based on the best procedure and those obtained by the other selected procedures described in section Supporting information (S1 Table) range from 0.9315 to 0.9980. As can be seen in Table 9, there are no major differences between rankings compared to the one created by the optimal procedure no. 120. This does not mean that all results of linear ordering are equally good. In the case of MDS procedures where the errors are not uniformly distributed, the aggregate measure, used to conduct linear ordering, can be concentrated or dispersed. Table 10 presents the percentage of errors associated with the arrangement of the three worst-performing objects for the selected procedures (with 31 objects analysed in the study, in the case of the uniform distribution, the share of errors for these three objects is equal to 9.677%).

**Table 10:** The percentage of errors associated with the arrangement of the three worst-performing objects for the selected procedures.

Procedure	*HHI*	Error per one object in % (uniform distribution)	Three objects with the highest error	Sum of errors in %
120	377.35	3.226	P, BG, CY	17.15
123	382.75	3.226	P, BG, EL	18.15
30	447.22	3.226	ES, MT, CZ	20.11
99	467.31	3.226	EL, CY, ES	24.00
100	505.56	3.226	CY, CZ, HU	27.01
59	519.75	3.226	ES, RO, EL	28.05
2	617.32	3.226	RO, CY, ES	33.05
1	618.89	3.226	RO, ES, CY	33.18
138	1621.45	3.226	P, AP, RO	64.17
137	1661.29	3.226	P, AP, RO	64.99

Source: calculated using the R [57].

**Fig 6:**
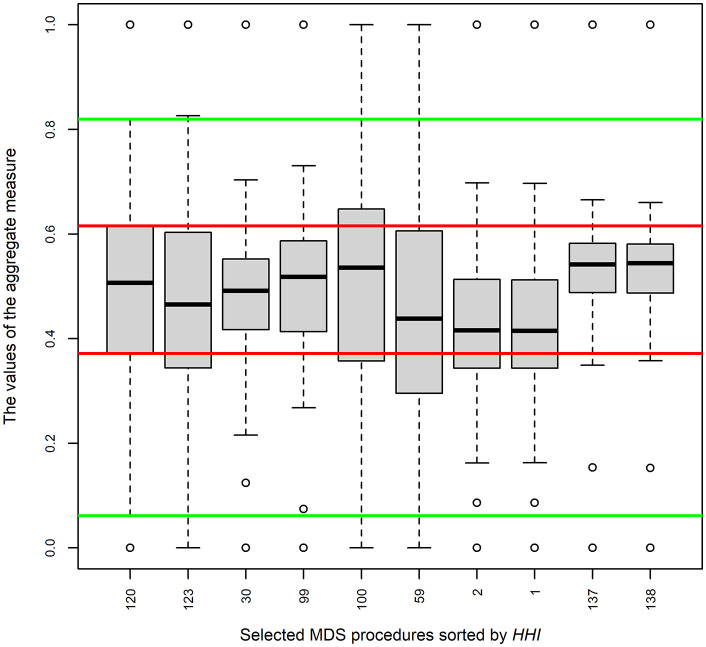
Boxplots for values of the aggregate measure for selected MDS procedures. Source: chart created using R [57].

Major differences in the value of the aggregate measure for the selected procedures are revealed by box plots in Fig 6. The 1^st^ and 3^rd^ quartiles for procedure 120 are marked in red, while green values represent the upper and the lower whiskers for the optimal procedure no. 120.

In the case of procedures whose error distributions deviate considerably from the uniform distribution (1, 2, 30, 99, 137, 138) the box plots are flatter than the box plot for the optimal procedure 120. For two procedures whose error distributions deviate considerably from the uniform distribution (59, 100), the plots have longer boxes and longer whiskers, which indicates a greater dispersion of the aggregate measure compared to that for procedure 120. These procedures yield considerably distorted results of linear ordering in terms of the aggregate measure.

Slight differences in the results of linear ordering relative to the optimal procedure were obtained for procedure 123, the second best in terms of the *HHI* index (normalization method: n11, distance measure: GDM1, scaling model: ratio). The corresponding box plot is not much different from that for procedure 120. Moreover, as can be seen in Table 9, the value of Spearman’s ρ, representing the rank correlation between the ranking created by procedure 120 and that obtained by procedure 123, is very high and equal to 0.9887.

## 7. Conclusions, discussion, and limitations

Since September 2015, 193 UN countries have been working to implement the resolution “Transforming our world: the 2030 Agenda for Sustainable Development” containing 17 Sustainable Development Goals (SDGs). To ensure sustainable performance in the context of the challenges posed by the 2030 Agenda, each EU country needs to properly assess its progress towards achieving SDGs. Given the importance of this need, we set out to analyse the performance of EU countries in relation to the core targets covered by SDG 4, measured by six indicators. The main aim of the study was to rank the 27 EU countries in terms of their progress towards SDG 4 in 2023 and to determine their distance of individual EU countries towards the 2030 target was also determined. By applying the proposed procedures, it was possible to divide the countries into five relatively homogenous classes with very high, high, middle, weak and very weak levels of progress towards SDG 4.

### 7.1. Discussion of the results in light of the literature

Progress across multiple SDGs is typically measured by means of aggregate measures. The main weakness of methods based on aggregate measures is their bias resulting from the compensation effect, whereby high performance on one indicator offsets poor performance on another – thereby potentially distorting policy conclusions. To overcome the compensation effect associated with the first and second approach, we proposed an innovative third approach, which accounts for the target values of the indicators and uses adjusted data (see section 2.2).

The study, to the best of the authors’ knowledge, represents the first attempt to assess progress towards the EU targets for 2030 with respect to SDG 4 in 2023. The application of the MDS procedure made it possible to visualise results of linear ordering in a two-dimensional space. The graphical presentation was enriched by the inclusion of development isoquants (curves of equal development) and a development path (i.e., the shortest line connecting the pattern and the anti-pattern object of development). This method of data visualization cannot be used with other linear ordering methods.

Our results suggest that progress towards SDG 4 in the EU is uneven across countries. While Northern and Western European countries are approaching the education targets for 2030, Southern and Eastern European member states continue to face persistent barriers. Taking into account values of the aggregate measure $d_{i\text{2}\text{0}\text{2}\text{3}}$ the top of the ranking is occupied mainly by the Nordic countries, with Finland holding the first place, followed by Netherlands, Denmark, Sweden and Estonia. However, even these countries face challenges in achieving SDG 4. Finland’s leading position reflects decades of systemic investment in equitable and high-quality schooling, extensive teacher training, and the integration of lifelong learning and digital competencies [58,59]. Finnish education policy, designed to minimize social stratification, demonstrates the effectiveness of treating education as a public good. Furthermore, Finland’s sustained investment in digital competencies and lifelong learning aligns closely with the two targets for SDG 4 (Adult participation in learning in the past four weeks and Share of individuals having at least basic digital skills), explaining its proximity to the EU’s 2030 education goal.

By contrast, according to our results, Romania and Bulgaria demonstrate the greatest distance from the 2030 target owing to structural barriers in education systems, including limited public expenditure on education, high regional disparities in early childhood and tertiary education, and weak adult learning participation rates. These challenges are further exacerbated by demographic decline, labour migration, and the persistent digital divide [60,61]. Such multidimensional constraints correspond to what Banerjee and Duflo [5] describe as “poverty traps,” in which underinvestment in education perpetuates cycles of low productivity and social exclusion. OECD [11] reports similarly highlight that countries with lower education expenditure and weaker institutional frameworks tend to lag in SDG 4 implementation.

Our findings suggest that the largest group includes 13 countries with a similar level of progress, as represented by an average level of the aggregate measure. Countries such as France, Portugal, and Poland display moderate performance, often achieving targets related to tertiary attainment and early school leaving, yet underperforming in adult learning and digital skills. This outcome underscores the non-compensatory nature of sustainable development progress, as advances in one dimension of education cannot offset deficiencies in others [15,22,39].

The findings of this study can also be interpreted through the lens of Doughnut Economics [62]. In this model, education represents one of the twelve dimensions of the social foundation, which together define the minimum social standards necessary for human well-being, as codified in the SDGs. Countries such as Finland and the Netherlands can be viewed as having secured the educational dimension of the social foundation while maintaining economic and environmental balance – remaining within what Raworth terms the “safe and just space for humanity.” Conversely, countries like Romania and Bulgaria remain below this social threshold, reflecting structural deficits in human capital and limited capacity to deliver equitable learning opportunities. The aggregate measure $d_{i\text{2}\text{0}\text{2}\text{3}}$ developed in this study can therefore serve as a diagnostic instrument for assessing how close or distant EU member states are from achieving the education-related social foundation within the broader sustainability framework.

### 7.2. Limitations

This study, while methodologically innovative, faces limitations. The main one was data availability. Data for the variable “Low achieving 15-year-olds in reading, mathematics or science” were only available for 2022 since they came from the PISA study, which is conducted every 3 years (for Luxembourg – only for 2018). Data for the variable “Participation in early childhood education” for Greece were only available for 2019.

Another limitation results from the selection of variables. The analysis is based on six Eurostat indicators, which primarily capture quantitative aspects of education systems. Therefore, important qualitative dimensions—such as teaching quality, curriculum relevance, and socio-emotional competencies—are not directly included in the analysis. Consequently, the proposed measure does not fully reflect the complexity of the concept of “quality education”.

Accordingly, the aggregate measure should be interpreted primarily as a screening and diagnostic tool for identifying potential areas of risk or lagging performance, rather than as a definitive measure of the overall quality of education systems.

### 7.3. Policy and research implications, future work

From an analytical standpoint, the study demonstrates the usefulness of the hybrid MDS-linear ordering approach in overcoming the compensation bias of traditional composite indicators. The results of multivariate analysis can be presented graphically in a two-dimensional space. This methodological innovation enhances transparency and comparability, offering a replicable model for evaluating other SDGs.

From a policy perspective, the proposed approach enables not only the ranking of countries but also the identification of specific patterns of strengths and weaknesses. In particular, the position of a country relative to the set axis and isoquants may support more precise policy interpretation. For example, a country located relatively close to the set axis but remaining distant from the target value due to weaker performance with respect to a specific group of indicators (such as adult learning participation or digital skills) may require targeted policy interventions focused on these areas. In contrast, countries located far from the target across multiple dimensions may require broader, cross-sectoral strategies addressing structural weaknesses in education systems, labour markets, and digital infrastructure. In practical terms, this means that policymakers can use the two-dimensional map to distinguish between countries with balanced but insufficient progress and those where a single indicator cluster is the main constraint, thereby prioritizing either focused or systemic interventions.

In theoretical terms, the results confirm that education plays a dual role as both a driver and an outcome of socio-economic development. The observed differences between EU countries indicate the need for further research taking into account institutional and structural factors.

Future research should focus on extending the analysis by incorporating micro-level data, longitudinal approaches, and qualitative dimensions of education, which would enable a more comprehensive understanding of SDG 4 implementation.

Calculations required in the hybrid approach described in the article are supported by the mdsOpt R package [44].

## Supporting information

S1 File Dataset_SDG 4_6 variables. (XLSX)

S2 File Data_P_AP. (CSV)

S3 File Data_P_AP_imput. (CSV)

S4 File Data_P_AP_imput_adjusted. (CSV)

S1 TableThe values of *Stress*-1 fit measure and the *HHI* index for p=144 MDS procedures.(DOCX)
