# Supplementary material for: Assessment of the implementation of SDG 4 goal by EU countries in the light of the 2030 Agenda using a hybrid approach in linear ordering
Source: PLoS One. 2026 Jun 22;21(6):e0333545. doi: 10.1371/journal.pone.0333545 (PMC13286218; doi:10.1371/journal.pone.0333545)
Supplement: S1 Table — (DOCX) [file pone.0333545.s005.docx]

**S1 Table. The values of *Stress*-1 fit measure and the *HHI* index for** $\boldsymbol{p=144}$ **MDS procedures**

| No | Normalization method | MDS model | Spline degree | Distance | *Stress-*1 | *HHI* |
| --- | --- | --- | --- | --- | --- | --- |
| 1 | n9a | mspline | 3 | euclidean | 0.061577 | 618.89 |
| 2 | n9a | mspline | 2 | euclidean | 0.061766 | 617.32 |
| 3 | n9a | interval |  | euclidean | 0.063332 | 602.81 |
| 4 | n9a | mspline | 3 | seuclidean | 0.064159 | 590.11 |
| 5 | n9a | mspline | 2 | seuclidean | 0.06585 | 585.04 |
| 6 | n9 | mspline | 3 | seuclidean | 0.06849 | 516.04 |
| 7 | n9 | mspline | 3 | euclidean | 0.068815 | 551.79 |
| 8 | n9 | mspline | 2 | euclidean | 0.068879 | 553.18 |
| 9 | n9 | mspline | 2 | seuclidean | 0.070026 | 513.58 |
| 10 | n9 | interval |  | euclidean | 0.071344 | 563.98 |
| 11 | n11 | mspline | 3 | seuclidean | 0.072896 | 495.22 |
| 12 | n11 | mspline | 2 | euclidean | 0.073161 | 516.39 |
| 13 | n11 | mspline | 3 | euclidean | 0.073166 | 516.09 |
| 14 | n11 | mspline | 2 | seuclidean | 0.074279 | 490.7 |
| 15 | n11 | interval |  | euclidean | 0.076015 | 512.72 |
| 16 | n9a | ratio |  | euclidean | 0.077165 | 550.06 |
| 17 | n9a | mspline | 2 | GDM1 | 0.078906 | 553.35 |
| 18 | n9a | mspline | 3 | GDM1 | 0.078955 | 553.65 |
| 19 | n9a | mspline | 3 | manhattan | 0.07933 | 474.14 |
| 20 | n9a | mspline | 2 | manhattan | 0.079732 | 483.11 |
| 21 | n9a | interval |  | manhattan | 0.080927 | 485.3 |
| 22 | n9 | mspline | 3 | manhattan | 0.080991 | 456.99 |
| 23 | n9 | mspline | 2 | manhattan | 0.081475 | 465.35 |
| 24 | n11 | mspline | 3 | manhattan | 0.082456 | 452.06 |
| 25 | n9 | interval |  | manhattan | 0.082811 | 468.9 |
| 26 | n11 | mspline | 2 | manhattan | 0.083016 | 459.88 |
| 27 | n8 | mspline | 3 | euclidean | 0.084008 | 444.9 |
| 28 | n8 | mspline | 2 | euclidean | 0.084058 | 446.54 |
| 29 | n11 | interval |  | manhattan | 0.084392 | 464.65 |
| 30 | n8 | mspline | 3 | seuclidean | 0.084642 | 447.22 |
| 31 | n8 | mspline | 2 | seuclidean | 0.086355 | 446.71 |
| 32 | n9a | ratio |  | manhattan | 0.086793 | 490.52 |
| 33 | n9 | ratio |  | euclidean | 0.087352 | 522.71 |
| 34 | n8 | mspline | 3 | manhattan | 0.087848 | 433.6 |
| 35 | n8 | mspline | 2 | manhattan | 0.088605 | 436.17 |
| 36 | n9 | mspline | 2 | GDM1 | 0.088916 | 517.37 |
| 37 | n9 | mspline | 3 | GDM1 | 0.088938 | 516.87 |
| 38 | n8 | interval |  | euclidean | 0.089062 | 452.47 |
| 39 | n9 | ratio |  | manhattan | 0.089349 | 476.67 |
| 40 | n8 | interval |  | manhattan | 0.090771 | 444.05 |
| 41 | n11 | ratio |  | manhattan | 0.0913 | 472.64 |
| 42 | n5a | mspline | 2 | euclidean | 0.093865 | 517.55 |
| 43 | n3 | mspline | 3 | euclidean | 0.093948 | 493.14 |
| 44 | n3 | mspline | 2 | euclidean | 0.09396 | 494 |
| 45 | n5a | mspline | 3 | euclidean | 0.094287 | 526.01 |
| 46 | n11 | ratio |  | euclidean | 0.094669 | 499.82 |
| 47 | n5a | mspline | 3 | seuclidean | 0.094777 | 496.6 |
| 48 | n5a | mspline | 3 | manhattan | 0.095573 | 427.93 |
| 49 | n5 | mspline | 2 | euclidean | 0.095714 | 522.65 |
| 50 | n5 | mspline | 3 | euclidean | 0.095912 | 528.94 |
| 51 | n5a | mspline | 2 | manhattan | 0.096051 | 422.98 |
| 52 | n5 | mspline | 3 | manhattan | 0.096247 | 423.62 |
| 53 | n5 | mspline | 3 | seuclidean | 0.096417 | 506.21 |
| 54 | n1 | mspline | 2 | euclidean | 0.096612 | 495.14 |
| 55 | n3 | mspline | 3 | manhattan | 0.09674 | 448.07 |
| 56 | n12a | mspline | 3 | manhattan | 0.096899 | 442.56 |
| 57 | n5 | mspline | 2 | manhattan | 0.096945 | 420.6 |
| 58 | n12a | mspline | 2 | euclidean | 0.09708 | 493.19 |
| 59 | n11 | mspline | 3 | GDM1 | 0.097157 | 519.75 |
| 60 | n9a | interval |  | GDM1 | 0.097211 | 394.6 |
| 61 | n11 | mspline | 2 | GDM1 | 0.097309 | 524.05 |
| 62 | n12a | mspline | 2 | manhattan | 0.097429 | 436.68 |
| 63 | n12a | mspline | 3 | euclidean | 0.097515 | 501.56 |
| 64 | n1 | mspline | 3 | euclidean | 0.097526 | 503.74 |
| 65 | n3 | mspline | 2 | manhattan | 0.097774 | 446.27 |
| 66 | n1 | mspline | 3 | seuclidean | 0.097979 | 477.5 |
| 67 | n12a | mspline | 3 | seuclidean | 0.098238 | 476.27 |
| 68 | n5a | mspline | 2 | seuclidean | 0.098411 | 492.1 |
| 69 | n1 | mspline | 3 | manhattan | 0.098451 | 445.8 |
| 70 | n1 | mspline | 2 | manhattan | 0.098764 | 437.63 |
| 71 | n8 | ratio |  | manhattan | 0.099288 | 452.86 |
| 72 | n5 | mspline | 2 | seuclidean | 0.099412 | 500.03 |
| 73 | n5a | interval |  | manhattan | 0.09986 | 434.17 |
| 74 | n3 | interval |  | euclidean | 0.100864 | 473.29 |
| 75 | n12a | interval |  | manhattan | 0.100906 | 448.52 |
| 76 | n5 | interval |  | manhattan | 0.100955 | 431.23 |
| 77 | n3 | mspline | 3 | seuclidean | 0.101168 | 541.7 |
| 78 | n3 | interval |  | manhattan | 0.101426 | 461.14 |
| 79 | n5a | interval |  | euclidean | 0.101692 | 517.55 |
| 80 | n12a | mspline | 2 | seuclidean | 0.101896 | 472.15 |
| 81 | n1 | mspline | 2 | seuclidean | 0.101967 | 471.78 |
| 82 | n1 | interval |  | manhattan | 0.10263 | 452.46 |
| 83 | n3 | mspline | 2 | seuclidean | 0.10312 | 535.64 |
| 84 | n5 | interval |  | euclidean | 0.103435 | 512.65 |
| 85 | n9 | interval |  | GDM1 | 0.103502 | 406.34 |
| 86 | n1 | interval |  | euclidean | 0.105963 | 481.72 |
| 87 | n12a | interval |  | euclidean | 0.106057 | 481.32 |
| 88 | n11 | interval |  | GDM1 | 0.108663 | 419.03 |
| 89 | n5a | ratio |  | manhattan | 0.109297 | 451.43 |
| 90 | n5 | ratio |  | manhattan | 0.110504 | 451.56 |
| 91 | n3 | ratio |  | manhattan | 0.111416 | 467.17 |
| 92 | n12a | ratio |  | manhattan | 0.111756 | 461.56 |
| 93 | n1 | ratio |  | manhattan | 0.112002 | 464.02 |
| 94 | n8 | ratio |  | euclidean | 0.113022 | 448.22 |
| 95 | n8 | mspline | 3 | GDM1 | 0.118437 | 474.76 |
| 96 | n8 | mspline | 2 | GDM1 | 0.118602 | 475.34 |
| 97 | n8 | interval |  | GDM1 | 0.123876 | 423.32 |
| 98 | n3 | ratio |  | euclidean | 0.127106 | 441.34 |
| 99 | n5a | ratio |  | euclidean | 0.127375 | 467.31 |
| 100 | n3 | mspline | 3 | GDM1 | 0.12813 | 505.56 |
| 101 | n3 | mspline | 2 | GDM1 | 0.128666 | 505.03 |
| 102 | n5 | ratio |  | euclidean | 0.1291 | 458.23 |
| 103 | n3 | interval |  | GDM1 | 0.132222 | 462.71 |
| 104 | n5a | mspline | 2 | GDM1 | 0.132755 | 486.93 |
| 105 | n5a | mspline | 3 | GDM1 | 0.132768 | 488.41 |
| 106 | n12a | ratio |  | euclidean | 0.13339 | 443.32 |
| 107 | n1 | ratio |  | euclidean | 0.13345 | 442.87 |
| 108 | n5 | mspline | 3 | GDM1 | 0.134148 | 482.57 |
| 109 | n5 | mspline | 2 | GDM1 | 0.134267 | 482.13 |
| 110 | n5a | interval |  | GDM1 | 0.136183 | 444.42 |
| 111 | n5 | interval |  | GDM1 | 0.137018 | 443.66 |
| 112 | n12a | mspline | 3 | GDM1 | 0.139419 | 458.61 |
| 113 | n12a | mspline | 2 | GDM1 | 0.139498 | 457.19 |
| 114 | n1 | mspline | 3 | GDM1 | 0.139919 | 461.77 |
| 115 | n1 | mspline | 2 | GDM1 | 0.140009 | 459.5 |
| 116 | n12a | interval |  | GDM1 | 0.141998 | 428.47 |
| 117 | n1 | interval |  | GDM1 | 0.143121 | 429.97 |
| 118 | n3 | ratio |  | GDM1 | 0.152033 | 394.7 |
| 119 | n9a | interval |  | seuclidean | 0.155686 | 685.27 |
| 120 | n8 | ratio |  | GDM1 | 0.156543 | 377.35 |
| 121 | n9 | interval |  | seuclidean | 0.15663 | 696.6 |
| 122 | n11 | interval |  | seuclidean | 0.157934 | 711.43 |
| 123 | n11 | ratio |  | GDM1 | 0.1589 | 382.75 |
| 124 | n12a | ratio |  | GDM1 | 0.158945 | 399.62 |
| 125 | n5 | ratio |  | GDM1 | 0.158957 | 393.19 |
| 126 | n5a | ratio |  | GDM1 | 0.159344 | 390.54 |
| 127 | n9 | ratio |  | GDM1 | 0.159548 | 387.19 |
| 128 | n1 | ratio |  | GDM1 | 0.15984 | 399.64 |
| 129 | n9a | ratio |  | GDM1 | 0.162078 | 390.65 |
| 130 | n8 | interval |  | seuclidean | 0.163735 | 765.39 |
| 131 | n3 | interval |  | seuclidean | 0.164982 | 720.81 |
| 132 | n5 | interval |  | seuclidean | 0.167554 | 767.18 |
| 133 | n5a | interval |  | seuclidean | 0.169801 | 800.06 |
| 134 | n12a | interval |  | seuclidean | 0.171726 | 832.2 |
| 135 | n1 | interval |  | seuclidean | 0.172522 | 850.33 |
| 136 | n3 | ratio |  | seuclidean | 0.224197 | 1374.84 |
| 137 | n1 | ratio |  | seuclidean | 0.230089 | 1661.29 |
| 138 | n12a | ratio |  | seuclidean | 0.230361 | 1621.45 |
| 139 | n5 | ratio |  | seuclidean | 0.232519 | 1467.47 |
| 140 | n5a | ratio |  | seuclidean | 0.235551 | 1528.68 |
| 141 | n8 | ratio |  | seuclidean | 0.241021 | 1409.78 |
| 142 | n11 | ratio |  | seuclidean | 0.249858 | 1281.98 |
| 143 | n9 | ratio |  | seuclidean | 0.253318 | 1248.3 |
| 144 | n9a | ratio |  | seuclidean | 0.25751 | 1217.78 |

seuclidean – squared Euclidean distance.

Source: own presentation using R [57].
